# Quantitative Structural Thresholds for Blood–Brain Barrier Permeability Derived from Experimental logBB Measurements

**DOI:** 10.3390/pharmaceutics18080967

**Published:** 2026-08-06

**Authors:** Saurabh Tiwari, Katarzyna Mądra-Gackowska, Marcin Gackowski, Łukasz Szeleszczuk

**Affiliations:** 1 School of Materials Science and Engineering, Yeungnam University, Gyeongsan 38541, Republic of Korea; 2 Department of Geriatrics, L. Rydygier Collegium Medicum in Bydgoszcz, Nicolaus Copernicus University in Torun, 9 Skłodowskiej Curie Str., 85-094 Bydgoszcz, Poland; katarzyna.madra@cm.umk.pl; 3 Department of Toxicology and Bromatology, L. Rydygier Collegium Medicum in Bydgoszcz, Nicolaus Copernicus University in Torun, 2 Jurasza Str., 85-089 Bydgoszcz, Poland; marcin.gackowski@cm.umk.pl; 4 Department of Organic and Physical Chemistry, Medical University of Warsaw, 1 Banacha Str., 02-097 Warsaw, Poland; lukasz.szeleszczuk@wum.edu.pl

**Keywords:** blood–brain barrier, logBB, BBB permeability, TPSA, CNS MPO, decision tree, scaffold analysis, rule-based prediction, B3DB

## Abstract

**Background/Objectives:** Rules for predicting blood–brain barrier (BBB) permeability, including the CNS multiparameter optimization (CNS MPO) score, Lipinski’s Rule of Five, and Veber’s rules, were developed using relatively limited datasets and have not been systematically re-evaluated using modern large-scale experimental databases. Using the B3DB database, which contains 1058 experimentally measured logBB values, we derived quantitative, data-driven structural thresholds for BBB permeability and benchmarked them against established heuristic rules. **Methods:** Six key physicochemical properties were calculated for all compounds, and optimal classification thresholds were identified through exhaustive optimizations. Decision trees and scaffold analyses were used to generate interpretable medicinal chemistry guidelines. **Results:** The topological polar surface area (TPSA) emerged as the strongest single predictor of BBB permeability, with an optimal threshold of 66.8 Å^2^ (AUC = 0.731, 95% CI: 0.689–0.771). This threshold outperformed the approximated CNS MPO ≥ 4 (AUC = 0.625), Lipinski’s Rule of Five (AUC = 0.546), and Veber rules (AUC = 0.566). A simple two-parameter rule combining TPSA < 67 Å^2^ and H-bond donors ≤ 1 achieved 96.6% precision for BBB-permeable compounds while maintaining an AUC of 0.720. Decision tree analysis further confirmed TPSA as the dominant determinant of BBB permeability, whereas scaffold analysis identified the molecular frameworks associated with highly permeable and impermeable compounds. External validation provided preliminary support for the improved specificity of the proposed rule, compared with existing approaches. **Conclusions:** These findings suggest that the commonly applied TPSA threshold of 90 Å^2^ may be overly lenient. A data-driven threshold of approximately 67 Å^2^ substantially improved the discrimination of BBB permeability across the entire dataset. Compounds with TPSA values between 67 and 90 Å^2^ should be assessed on a case-by-case basis, considering the ionization state and active transport potential, rather than being automatically classified as BBB-permeable. These experimentally grounded rules offer a practical framework for the early-stage design of CNS leads.

## 1. Introduction

Blood–brain barrier (BBB) penetration is a prerequisite for central nervous system (CNS) drug activity and a persistent bottleneck in CNS drug discovery. The BBB is constituted at the level of brain capillary endothelial cells, which are connected by tight junctions and form the primary anatomical barrier. Pericytes and astrocytic end-feet are closely associated with the abluminal surface and play essential roles in maintaining barrier integrity through paracrine signaling, whereas the selective barrier itself resides in the endothelial cell layer [1,2,3]. This interface excludes approximately 98% of small molecules from the brain parenchyma. Tight junctions between endothelial cells, combined with efflux transporters such as P-glycoprotein (P-gp) and breast cancer resistance protein (BCRP), further restrict the passive and active entry of xenobiotics into the brain. Therefore, BBB permeability is a primary objective in the design of CNS-targeted compounds. Conversely, for drugs intended for peripheral targets, CNS exclusion reduces the risk of CNS-mediated adverse effects, such as sedation, cognitive impairment, and seizures. Quantitative BBB permeability is most described by the brain-to-blood concentration ratio at steady state, expressed as logBB = log(Cbrain/Cblood). Compounds with logBB > 0 are considered highly CNS-penetrant, those with −1.0 ≤ logBB ≤ 0 are moderately penetrant, and logBB < −1.0 indicates effective BBB exclusion [4,5,6]. Although logBB is a simplification that does not distinguish between passive permeability and active transport contributions, it remains the most widely used single metric for BBB characterization in computational studies owing to its availability in curated databases and its direct relevance to CNS exposure in animal models.

Early efforts to predict BBB permeability relied on physicochemical property rules derived from relatively small sets of compounds. Lipinski’s Rule of Five (Ro5) [7], originally developed to predict oral absorption and bioavailability, is frequently applied to CNS compounds by proxy because intestinal permeability and passive CNS penetration share overlapping physicochemical requirements. However, Ro5 was developed for oral bioavailability rather than BBB penetration, and its application to CNS compound filtering represents a secondary use, whose empirical predictivity for logBB is evaluated in Section 3.5. CNS drugs often occupy a narrower physicochemical property space than oral drugs in general [8]. Similarly, Veber et al. demonstrated that rotatable bonds ≤ 10 and TPSA ≤ 140 Å^2^ reliably predict oral bioavailability in rats; these thresholds were likewise derived for intestinal absorption rather than brain penetration [9], and their empirical predictivity for logBB is evaluated in this study. The most CNS-specific heuristic framework is the CNS multiparameter optimization (CNS MPO) score, introduced by Wager et al. [8,10], which was developed as a multidimensional CNS drug optimization framework rather than as a dedicated logBB classifier. The CNS MPO integrates six desirability functions, namely CLogP, MW, TPSA, H-bond donors (HBD), logD at pH 7.4, and pKa of the most basic center into a composite score ranging from 0 to 6. Compounds with scores ≥ 4 were associated with favorable CNS drug properties in a proprietary Pfizer dataset. The CNS MPO has been widely adopted in industrial CNS drug discovery as a holistic design scorecard, and retrospective analyses have shown that marketed CNS drugs cluster at CNS MPO ≥ 4 (approx.) more often than non-CNS drugs [8,10,11]. Nevertheless, the score was calibrated on proprietary data, its six component thresholds were set by expert judgment rather than optimization against experimental logBB measurements, and its predictive performance against large independent experimental databases has not yet been systematically evaluated.

A longstanding challenge in BBB prediction is the limited size of publicly available experimental data. Until recently, most published models were trained on datasets of 100–400 compounds, raising concerns regarding generalizability across the chemical space [4]. The B3DB database, introduced by Meng et al. in 2021 [12], substantially changed this landscape by curating 7807 binary BBB+/BBB− classifications and 1058 quantitative logBB measurements from the primary literature, which is the largest and most chemically diverse experimental BBB dataset available to date. The B3DB spans a wide range of therapeutic classes, including central nervous system (CNS) drugs, antibiotics, antivirals, antineoplastics, and cardiovascular agents, providing unusually broad chemical coverage for threshold analysis.

Machine learning models trained on B3DB have demonstrated substantially improved performance compared to those trained on earlier datasets. A recent machine-learning study using the same B3DB dataset [13] showed that a gradient boosting classifier trained on 40 Mordred molecular descriptors achieved AUC = 0.9476 on the internal test set and AUC = 0.9137 on an independent external validation set of 175 compounds. Shapley additive explanation (SHAP) analysis identified the topological polar surface area (TopoPSA/TPSA) as the dominant predictor, with a mean absolute SHAP value of 0.318, which was approximately 2.4-fold higher than that of the second-ranked descriptor (SLogP, mean |SHAP| = 0.134). This finding raises an important follow-up question that ensemble models cannot answer directly: if TPSA is the dominant predictor, what is the optimal experimental TPSA threshold derived from the full B3DB dataset, and how does it compare with the thresholds embedded in existing heuristic rules?

This study addresses this question by applying interpretable analytical methods, including exhaustive threshold optimization, limited depth decision trees, and Murcko scaffold analysis, to the B3DB regression subset (*n* = 1058) data. Unlike ensemble machine learning models, which sacrifice transparency for predictive accuracy, decision trees produce explicit IF-THEN rules that medicinal chemists can apply by inspecting the structure. The tradeoff in AUC between a depth-4 decision tree and a gradient boosting model was well-characterized: approximately 0.20 AUC units, in exchange for complete mechanistic transparency and no computational requirements. Both types of models have distinct practical values, and the rules derived here are designed to complement, not replace, the quantitative predictions of our companion study. The specific aims of this study are as follows: (1) to derive optimal single-feature and two-feature BBB classification thresholds from B3DB experimental logBB data; (2) to benchmark data-driven rules against CNS MPO ≥ 4, (approx.) Lipinski Ro5, and Veber rules on both the full B3DB dataset and an independent external set; (3) to visualize the physicochemical property space separating BBB+ and BBB− compounds; (4) to identify BBB-permeable and BBB-impermeable Murcko scaffolds enriched in the B3DB dataset; and (5) to provide decision-tree–based IF-THEN rules with quantitative thresholds suitable for immediate use in CNS compound design.

## 2. Materials and Methods

### 2.1. Dataset

The B3DB database (version 1.1.1, qc-B3DB Python package) was used as the primary data source [12]. The regression subset contained 1058 compounds with experimentally measured logBB values (mean = −0.078, SD = 0.751, range: −2.69 to +1.70). An external classification set (*n* = 175; 171 BBB+, 4 BBB−) was used for external validation. All SMILES strings were validated using RDKit (version 2026.3.2), and all 1058 compounds passed structure parsing without modification of the strings. Binary BBB classification was defined as BBB+ if logBB ≥ −1.0 and BBB− if logBB < −1.0 [4]. This threshold yielded 930 BBB+ (87.9%) and 128 BBB− (12.1%) compounds in the regression set, consistent with the known enrichment of BBB-permeable compounds in pharmacological databases (Supplementary Table S1).

#### Data Curation and Preprocessing

The B3DB v1.1.1 dataset was used without additional filtering beyond SMILES validity, with all 1058 compounds successfully parsed using RDKit. Data preprocessing followed the original B3DB curation protocol [12]: (a) salts and counterions only parent structures were retained following salt stripping; (b) stereoisomers not distinguished, with the mean logBB recorded when multiple stereoisomers were reported; (c) tautomers canonical tautomers assigned during B3DB curation; (d) multiple logBB measurements the mean experimental logBB value was used for each compound; (e) species the dataset consists predominantly of rat in vivo logBB measurements, while human data represent a minority and are not separately identified in B3DB v1.1.1; and (f) brain-to-blood versus brain-to-plasma measurements both measurement types are included without separate annotation, and this heterogeneity is acknowledged as a source of variability. No additional compound-level deduplication was performed beyond the original B3DB curation process. The exact binomial 95% confidence interval for the internal specificity estimate (*n* = 128 BBB− compounds; observed specificity = 0.852) was 0.775–0.912.

### 2.2. Descriptor Computation

Six physicochemical properties were computed for all compounds using RDKit (v2026.3.2) and Mordred (v1.2.0): (1) topological polar surface area (TPSA, Å^2^), (2) molecular weight (MW, Da), (3) calculated logP (CLogP), (4) H-bond donors (HBD), (5) H-bond acceptors (HBA), and (6) rotatable bonds (nRotB). In addition, 35 Mordred/RDKit descriptors were computed for decision tree analysis. All descriptor definitions and source references are provided in Supplementary Table S8 of the Supporting Information.

### 2.3. Optimal Threshold Identification

For each of the six key physicochemical properties, optimal classification thresholds were identified by an exhaustive search over the 5th–95th percentile range (1-percentile steps). For each candidate threshold and direction (≤ or >), the AUC-ROC was computed against the binary BBB classification (logBB ≥ −1.0). Bootstrap 95% confidence intervals (2000 resamples, seed = 42) were computed for all AUC values. The threshold that maximized the AUC was selected as the optimal B3DB-derived cutoff value. Two-feature rules were evaluated by combining individually optimal thresholds using the AND operator. To estimate optimism in the threshold selection procedure, a nested bootstrap analysis was performed using 1000 bootstrap resamples of the training set (*n* = 740), stratified by BBB class. Within each resample, the complete threshold-selection procedure was repeated, and the performance was evaluated on the corresponding out-of-bag samples. The median optimal TPSA threshold across bootstrap resamples was 70.4 Å^2^ (IQR: 65.3–81.7). The bias-corrected out-of-bag AUC was 0.736 (95% CI: 0.669–0.794), compared with a full-dataset AUC of 0.746, indicating only modest optimism with an absolute difference of 0.010.

### 2.4. Decision Tree Model Development

Decision tree models were trained using scikit-learn (v1.8.0) with hyperparameters specified a priori to maximize interpretability: a maximum depth of four (limiting rules to no more than four decision conditions) and a minimum of 15 samples per leaf (preventing trivially small terminal nodes). These hyperparameters were not optimized using a validation set. The data were partitioned into training, validation, and test sets (70/10/20; random seed = 42), stratified by logBB quintiles and consistent with the companion study [13]. The validation set (*n* = 106) was retained solely for consistency with that study and was not used for hyperparameter optimization; all reported model performances were evaluated on the held-out test set (*n* = 212). The 35 Mordred/RDKit descriptors were inherited from a companion study [13], where they were identified using SHAP importance analysis and were not re-selected in the present study. TPSA (RDKit) and TopoPSA (Mordred) were both calculated using the Ertl topological polar surface area algorithm and were numerically identical in this dataset (Pearson *r* = 1.000, *n* = 1058). Their appearance at different tree depths reflects minor floating-point implementation differences between the software libraries rather than independent structural information. Model stability was assessed using 5-fold stratified cross-validation (seed = 42) on the training set. The model performance was evaluated using R^2^ and RMSE for regression and AUC-ROC for classification.

### 2.5. Benchmark Rule Implementation

Six rule-based approaches and two baseline classifiers were implemented and evaluated using both the full B3DB dataset (*n* = 1058) and an external validation set (*n* = 175). A prevalence-based classifier (always predicting BBB+) was used as a reference baseline. In addition, a logistic regression model using TPSA and CLogP as predictors (scikit-learn LogisticRegression, default settings; same 70/10/20 split, seed = 42) was included as a baseline parametric model. Label-permutation testing (2000 stratified permutations) was also performed and confirmed above-chance performance for all reported rule-based approaches (permuted-label AUC = 0.500 ± 0.017). The CNS multiparameter optimization (CNS MPO) score proposed by Wager et al. [8,10] was implemented as an approximation, hereafter referred to as CNS MPO (approx.), because experimental logD and pKa values were unavailable for all compounds. Accordingly, logD at pH 7.4 was approximated using CLogP, and the pKa contribution was fixed at 0.5. Compounds with CNS MPO (approx.) scores ≥ 4 were classified as BBB+. Sensitivity analysis confirmed that the fixed pKa assumption did not affect the binary AUC of the approximated implementation (Supplementary Note S1). Full implementation details are provided in Supplementary Note S1. Lipinski’s Rule of Five (Ro5) [7] was implemented using the conventional criteria of molecular weight ≤ 500 Da, CLogP ≤ 5, H-bond donors ≤ 5, and H-bond acceptors ≤ 10, with compounds satisfying all criteria classified as BBB+. Veber’s rules [9] were implemented using rotatable bonds ≤ 10 and TPSA ≤ 140 Å^2^. The B3DB-derived rule developed in this study classified compounds as BBB+ when TPSA was < 67 Å^2^ and the number of H-bond donors was ≤ 1.

### 2.6. Murcko Scaffold Analysis

Murcko scaffold frameworks were extracted using RDKit MurckoScaffold.MurckoScaffoldSmiles() with chirality excluded. Scaffolds with ≥3 representative compounds in the B3DB regression set were retained (*n* = 77 scaffolds). Mean logBB was computed per scaffold. Scaffolds enriched in BBB-permeable compounds were defined as those with a mean logBB ≥ 0, and scaffolds enriched in BBB-impermeable compounds were defined as those with a mean logBB ≤ −0.5. All 77-scaffold data are listed in Supplementary Table S5.

### 2.7. External Validation Strategy

All rules were independently evaluated using the B3DB external classification dataset (*n* = 175; 171 BBB+ and 4 BBB− compounds). As described by Meng et al. [12], external compound labels were assigned using the logBB ≥ −1.0 criterion when quantitative measurements were available, whereas consensus BBB+/BBB− classifications derived from DrugBank, ChEMBL, and pharmacokinetic literature were used when quantitative logBB values were unavailable. Therefore, the derived endpoint (quantitative logBB threshold) and the external evaluation endpoint (categorical BBB classification) are not fully equivalent. Accordingly, the external analysis should be interpreted as an assessment of rule transportability to external categorical classifications, rather than as a quantitative validation of the 66.8 Å^2^ threshold. The structural similarity between the training and external datasets was assessed using pairwise Tanimoto similarity based on Morgan fingerprints (radius = 2, 1024 bits). The mean maximum training–external similarity was 0.60 ± 0.27 (median = 0.61). The AUC, sensitivity, and specificity were calculated using external categorical labels. Bootstrap 95% confidence intervals for AUC were obtained using stratified resampling while maintaining the 171:4 BBB+− ratio in each resample to avoid the generation of samples lacking BBB− compounds. Because the external dataset contained only four BBB− compounds, specificity estimates were calculated with exact Clopper–Pearson 95% confidence intervals and should be interpreted cautiously. All analyses were performed using Python 3.12. Statistical comparisons were performed using the two-sided Mann–Whitney U test (two-sided).

## 3. Results

### 3.1. B3DB Dataset Characteristics

**Figure 1:**
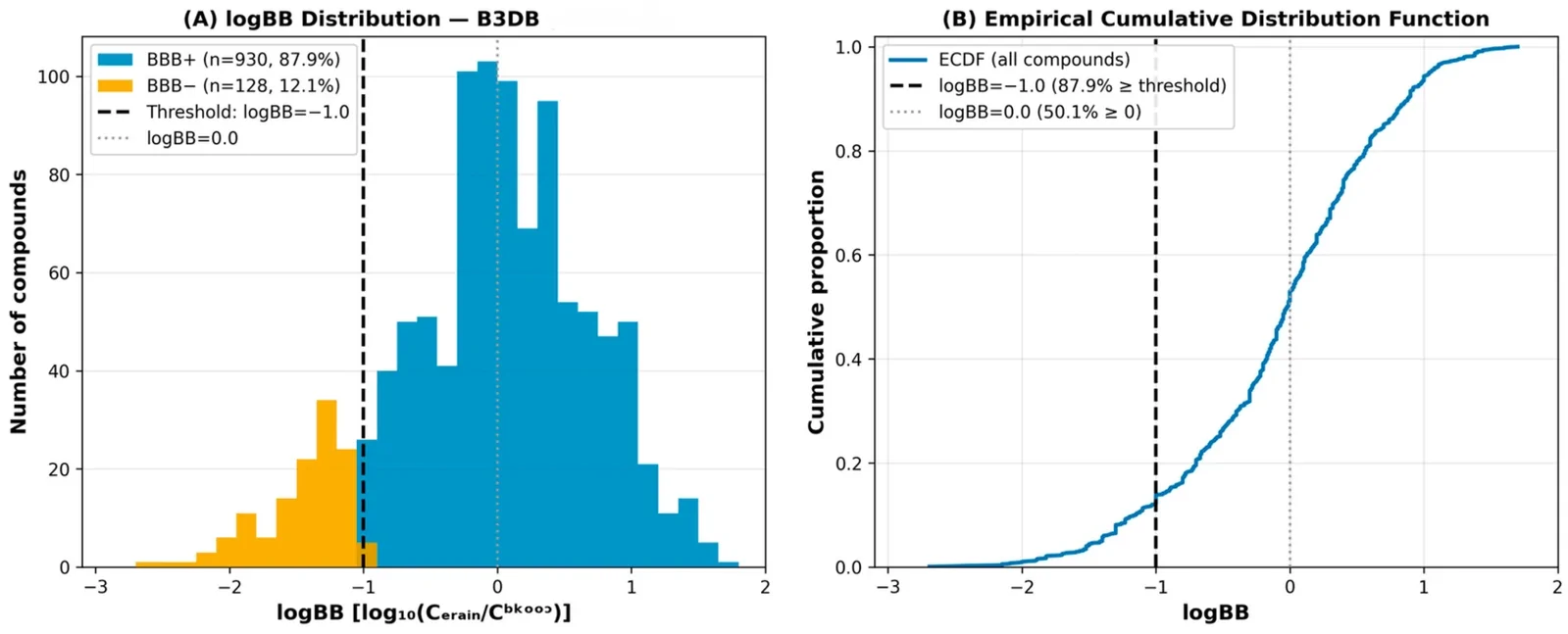
Distribution of logBB values in the B3DB regression dataset (*n* = 1058). logBB is defined as log_10_(Cbrain/Cblood), representing the total brain-to-blood concentration ratios compiled from predominantly rat in vivo studies in the B3DB. (**A**) Histogram showing BBB classification based on the conventional threshold of logBB = −1.0: BBB+ (blue, *n* = 930, 87.9%) and BBB− (orange, *n* = 128, 12.1%). The dashed line indicates the classification boundaries. (**B**) Empirical cumulative distribution function (ECDF) showing the cumulative proportion of compounds across the logBB values. The vertical dashed line at logBB = −1.0 indicates the BBB classification threshold.

The B3DB regression dataset (*n* = 1058) spanned logBB values from −2.69 to +1.70, with a median logBB of −0.020 (mean = −0.078, SD = 0.751). The distribution was approximately normal but slightly bimodal, with a concentration of compounds near logBB = 0 and a secondary cluster below −1.0 (Figure 1A). Applying the conventional logBB ≥ −1.0 threshold yielded 930 BBB+ (87.9%) and 128 BBB− (12.1%) compounds (Figure 1B). This pronounced class imbalance reflects the inherent enrichment of CNS-active compounds in pharmacological databases, where bioactive and CNS-penetrant molecules are over-represented.

### 3.2. Decision Tree Performance and Interpretation

**Figure 2:**
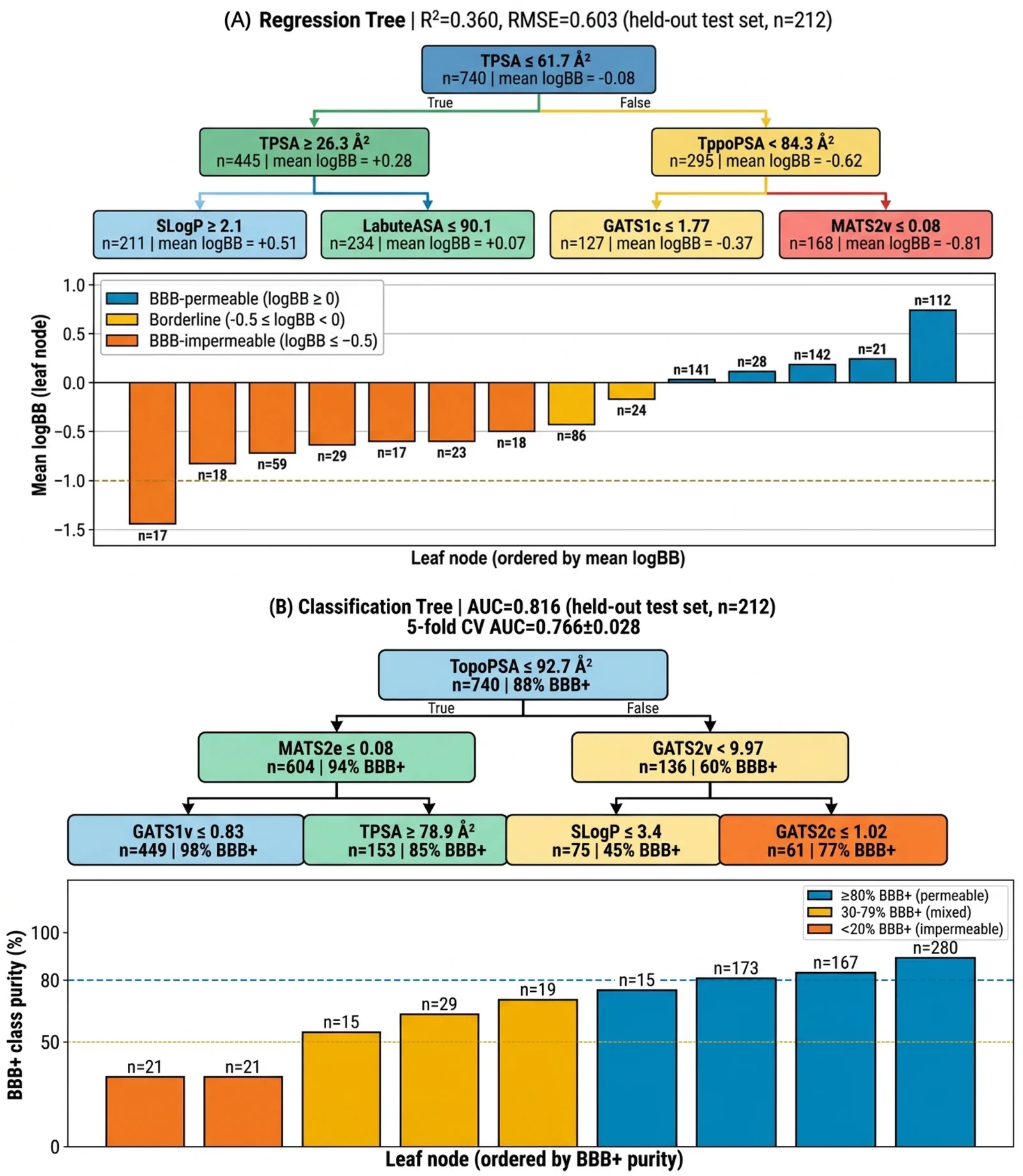
Decision tree models for logBB prediction and BBB classification. (**A**) Regression Decision Tree | depth = 4 | R^2^ = 0.360, RMSE = 0.603 (held-out test set, *n* = 212). (**B**) Classification Decision Tree | depth = 4 | AUC = 0.816 (held-out test set, *n* = 212); 5-fold cross-validation AUC = 0.766 ± 0.028. Both trees used the topological polar surface area (TPSA/TopoPSA) as the primary splitting variable. TPSA (RDKit) and TopoPSA (Mordred) were calculated using the same Ertl algorithm and were numerically identical in this dataset (Pearson r = 1.000); their occurrence at different tree depths reflects minor software implementation differences rather than independent structural information.

Decision trees (depth = 4) were trained on 35 Mordred descriptors using the same stratified 70/10/20 split as in our companion study [13] (740 train, 106 validations, 212 test; seed = 42). The regression decision tree achieved R^2^ = 0.360 (RMSE = 0.603) on the held-out test set; five-fold cross-validation yielded a mean AUC of 0.766 ± 0.028 (classification tree), confirming model stability. The classification decision tree achieved a test AUC of 0.816, indicating good performance. Both trees used TPSA (or TopoPSA) as the primary split variable, confirming the central role of the polar surface area in BBB permeability discrimination. The regression tree primary split was TPSA ≤ 61.7 Å^2^, and the classification tree used TopoPSA ≤ 92.7 Å^2^. The trees are shown in Figure 2 (descriptor abbreviations are defined in the Figure 2 caption, Table 1 legend, and Supplementary Table S8).

The key IF-THEN rules extracted from the regression tree are presented in Table 1. Compounds with TPSA ≤ 36 Å^2^, SLogP > 2.1, and Geary autocorrelation GATS2c > 1.06 showed a mean predicted logBB of +0.77 (strongly BBB-permeable), whereas compounds with TPSA > 85 Å^2^ and nBase > 1.5 had a mean predicted logBB of −1.37 (strongly BBB-impermeable). Across all 15 leaf nodes, the mean predicted logBB ranged from −1.37 to +0.77, spanning 2.15 logBB units.

### 3.3. Property Distributions: BBB+ vs. BBB−

**Figure 3:**
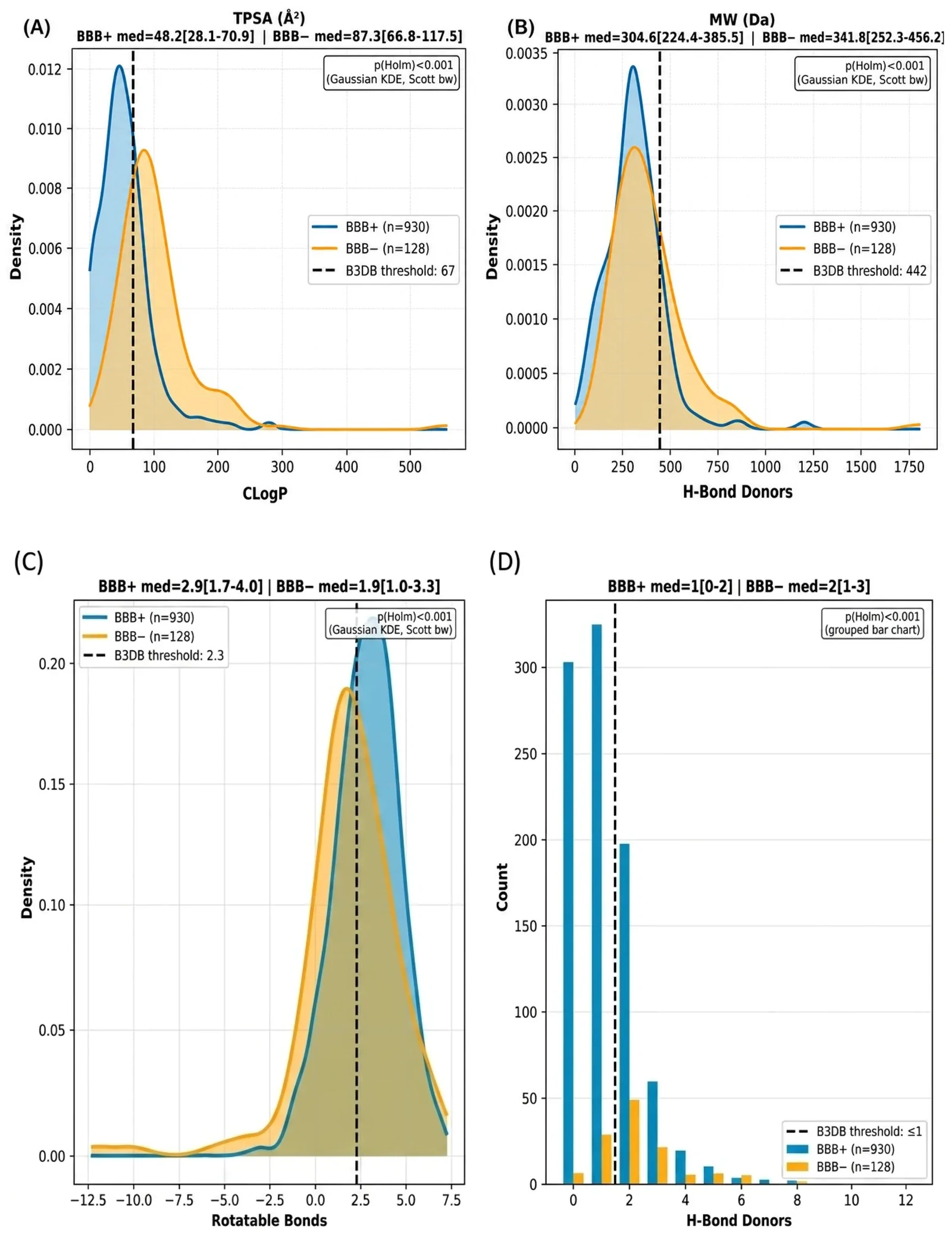

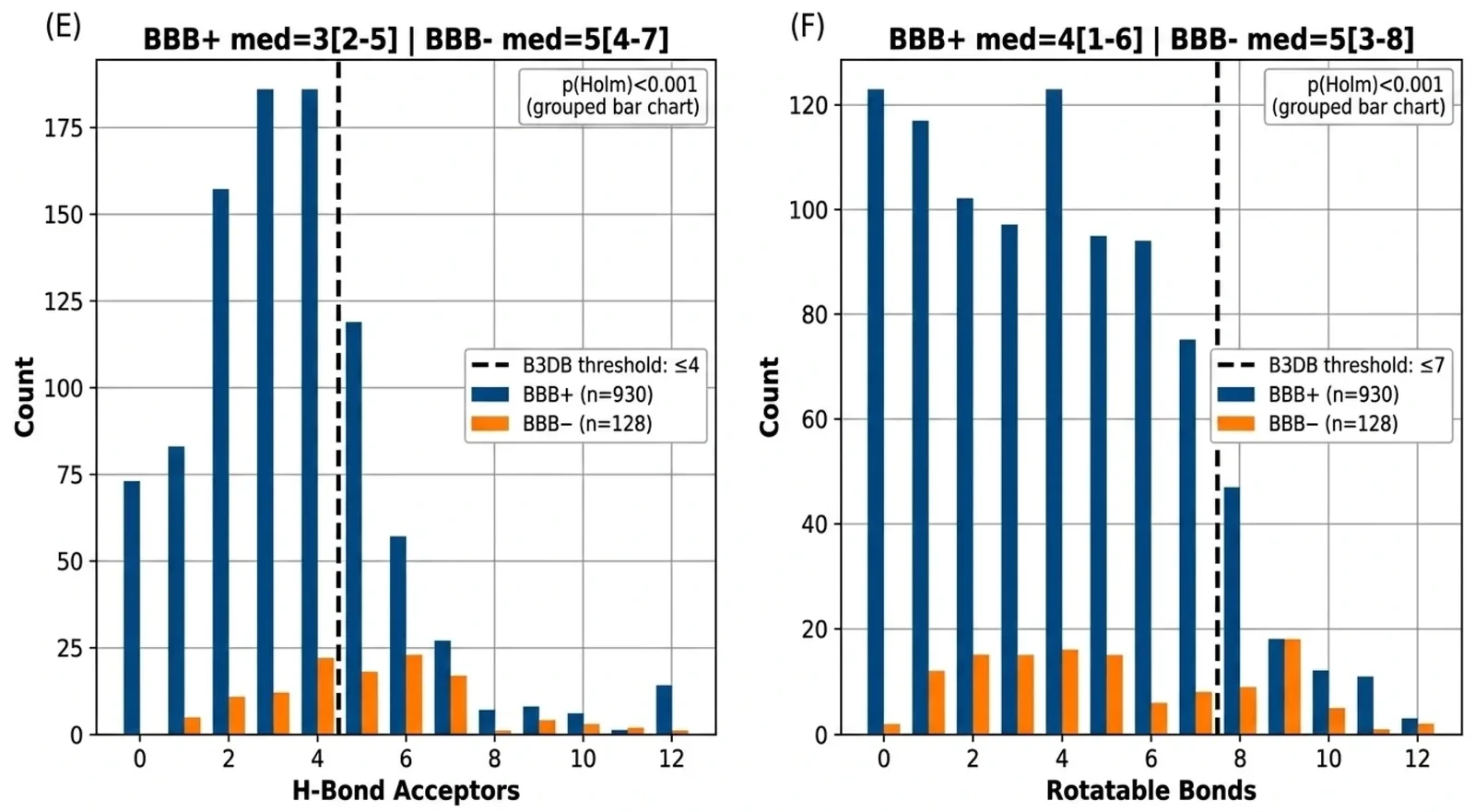
Physicochemical property distributions of BBB+ (blue) and BBB− (orange) compounds in the B3DB regression set (*n* = 1058). Vertical dashed lines indicate the B3DB-derived optimal thresholds. (**A**) TPSA, (**B**) molecular weight, (**C**) CLogP, (**D**) hydrogen bond donors, (**E**) hydrogen bond acceptors, and (**F**) rotatable bonds. The mean values are indicated in each panel.

Figure 3 shows the distributions of the six key properties of the BBB+ and BBB− compounds. The clearest separation was observed for TPSA (mean: BBB+ 53.9 ± 41.4 Å^2^ vs. BBB− 100.9 ± 62.7 Å^2^; *p* < 0.001) and HBD (mean: BBB+ 1.18 ± 1.22 vs. BBB− 2.48 ± 2.23; *p* < 0.001). CLogP showed the opposite trend (mean: BBB+ 2.82 ± 1.72 vs. BBB− 1.89 ± 2.62; *p* < 0.001), in line with the established role of lipophilicity in passive central nervous system (CNS) penetration. MW, HBA, and nRotB showed moderate but significant separation (all *p* < 0.001, Mann–Whitney U test). Effect sizes were evaluated using Cliff’s delta with 95% bootstrap confidence intervals (2000 resamples) and Holm-corrected *p*-values. The observed effect sizes were TPSA: δ = −0.595 [−0.671, −0.516]; HBD: δ = −0.488 [−0.571, −0.409]; HBA: δ = −0.458 [−0.552, −0.363]; MW: δ = −0.226 [−0.331, −0.123]; CLogP: δ = +0.246 [+0.133, +0.354]; and nRotB: δ = −0.254 [−0.364, −0.152]. Negative delta values indicated higher descriptor values in the BBB− group. All six comparisons remained statistically significant after Holm’s correction (all *p* (Holm) < 0.001). Grouped bar charts for the discrete variables (HBD, HBA, and nRotB) are presented in Figure 3D–F, and the medians and interquartile ranges are reported in Table 2. These observations are quantitatively summarized in Table 2.

### 3.4. Optimal Threshold Derivation

**Figure 4:**
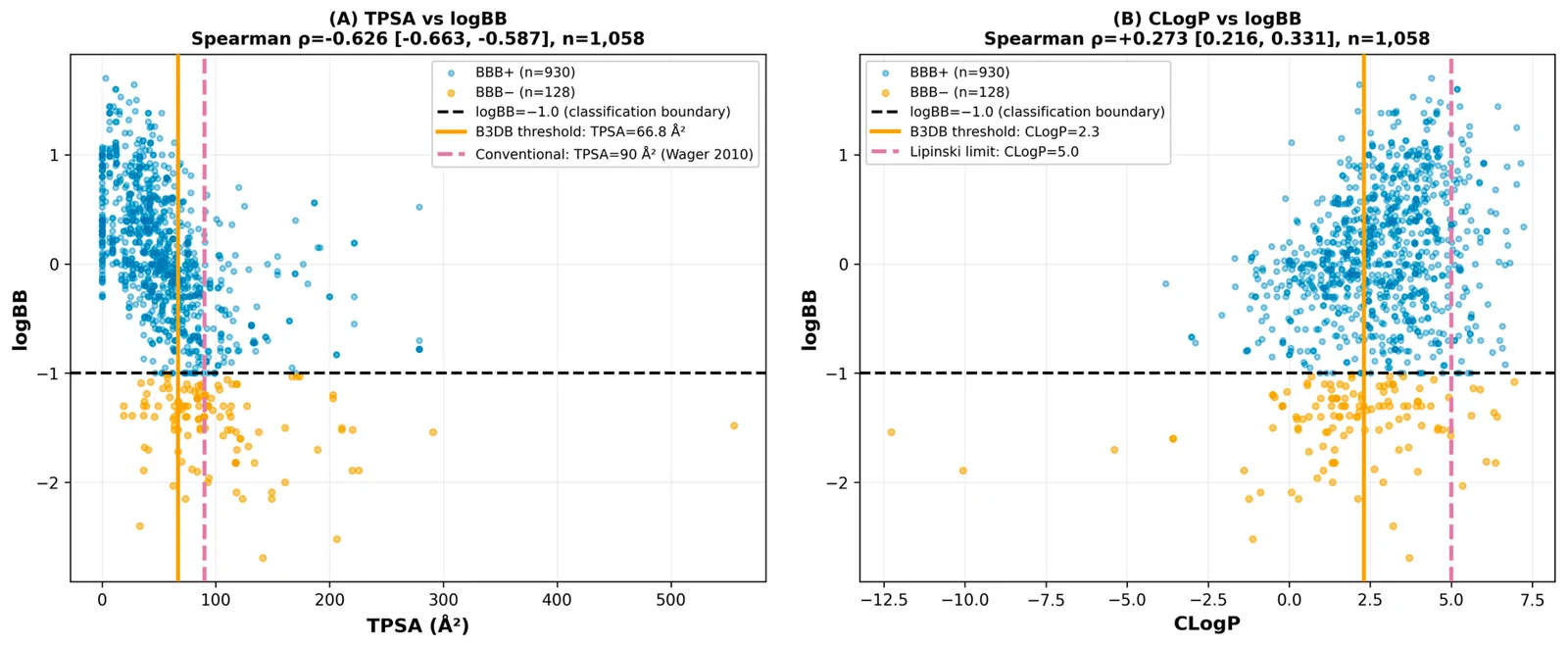
Relationship between logBB and key physicochemical properties in the B3DB dataset (*n* = 1058). (**A**) TPSA versus logBB (Spearman ρ = −0.626; 95% CI: −0.662 to −0.588). The B3DB-derived TPSA threshold of 66.8 Å^2^ (solid orange line) provides improved separation of BBB classes compared to the conventional TPSA threshold of 90 Å^2^ (dashed purple line). (**B**) CLogP versus logBB (Spearman ρ = +0.273; 95% CI: +0.215 to +0.330). The B3DB-derived CLogP threshold (>2.3; solid orange line) and conventional Lipinski CLogP limit (5; dashed purple line) are shown. The horizontal dashed line at logBB = −1.0 represents the BBB+/BBB− classification boundary and is not a threshold applied to the *x*-axis variables. BBB+ and BBB−compounds are shown in blue and orange, respectively.

Table 2 presents the B3DB-derived optimal thresholds for each property. The topological polar surface area emerged as the most predictive physicochemical property for BBB permeability (AUC = 0.746 [0.708–0.784]), exceeding the MW (AUC = 0.583), CLogP (AUC = 0.617), HBD (AUC = 0.699), HBA (AUC = 0.673), and nRotB (AUC = 0.596). The optimal TPSA cutoff of 66.8 Å^2^ is markedly lower than the 90 Å^2^ threshold of Wager et al. [8] and the 140 Å^2^ threshold of Veber et al. [9]. Scatter plots of TPSA and CLogP against logBB (Figure 4) confirm that the B3DB-optimized TPSA < 67 threshold achieves better visual separation of BBB classes than the conventional TPSA < 90 cutoff. The CLogP scatter plot revealed that although higher lipophilicity was associated with greater logBB, the relationship was noisier, explaining its lower single-feature AUC.

**Figure 5:**
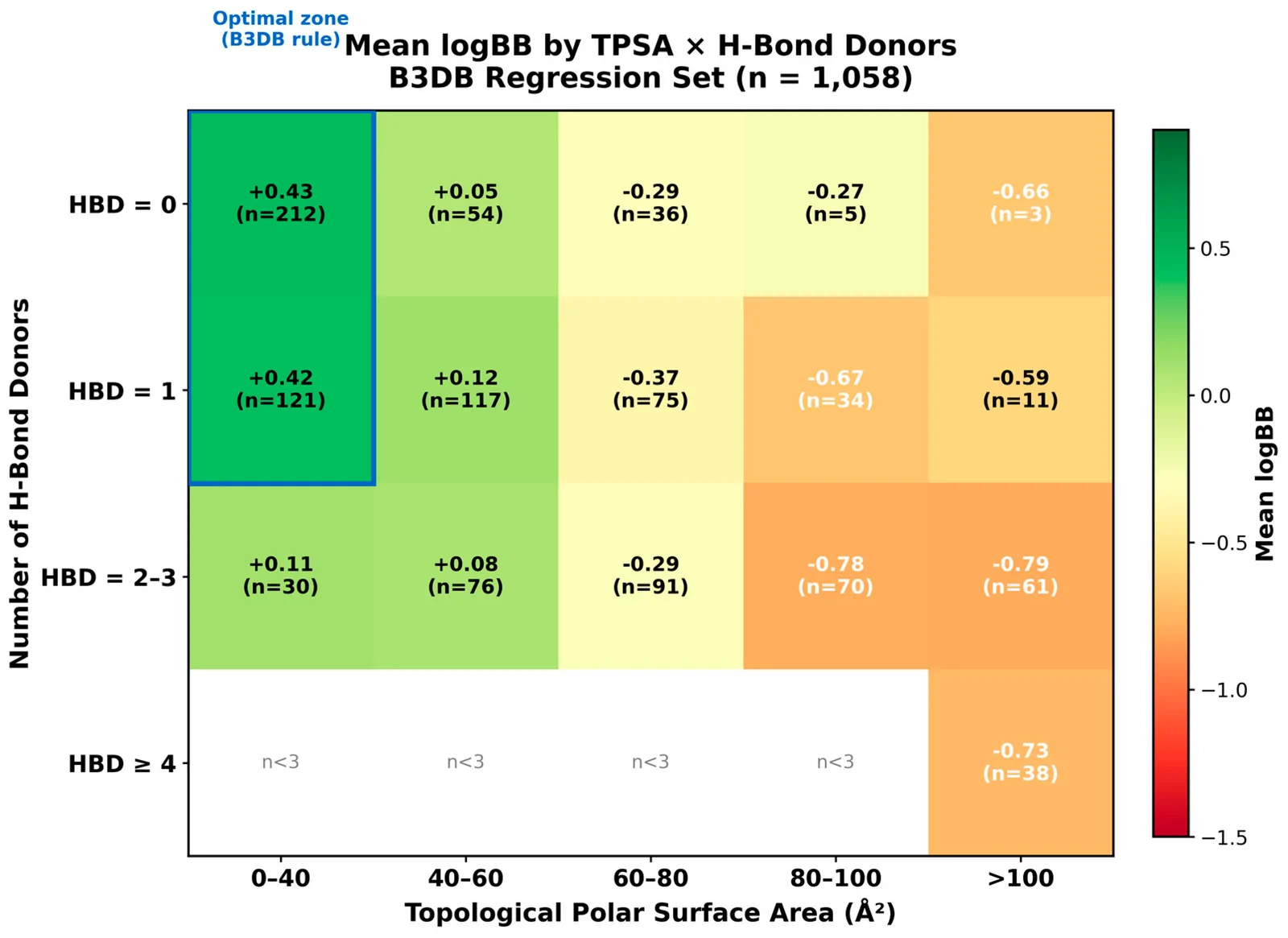
Heatmap of the mean logBB as a function of TPSA (*x*-axis, Å^2^) and H-bond donors (*y*-axis). The cell values indicate the mean logBB and compound count (*n*). Cells with *n* < 3 are shown as *n* < 3. The green-to-red color scale ranges from high (BBB-permeable) to low (BBB-impermeable) log BB values. The blue rectangle indicates the B3DB-derived optimal zone (TPSA 0–40 × HBD = 0, mean logBB = +0.43).

The heatmap of the mean logBB by combined TPSA × HBD bins (Figure 5) reveals a clear joint distribution pattern: the cell with the lowest TPSA (0–40 Å^2^) and HBD = 0 has a mean logBB of +0.43 (*n* = 212), while the cell with TPSA > 100 Å^2^ and HBD ≥ 4 has a mean logBB of −0.73 (*n* = 38). A logistic regression model including TPSA, HBD, and their interaction term (TPSA × HBD) produced no meaningful improvement in AUC compared with the additive model (AUC = 0.802 vs. 0.803), indicating no detectable interaction effects between these variables. The mean logBB reduction associated with adding one HBD at a constant low TPSA (0–40 bin) was approximately −0.01 logBB units (HBD = 0 to HBD = 1: +0.43 vs. +0.42), whereas increasing TPSA from 40 to 60 to 60–80 Å^2^ was associated with a reduction of approximately −0.34 to −0.49 logBB units. Therefore, reducing both TPSA and HBD is the most favorable strategy for increasing BBB penetration, with TPSA showing a larger associated effect across HBD levels. All heatmap source values are listed in Supplementary Table S6.

### 3.5. Comparative Performance of Rule-Based Approaches

**Figure 6:**
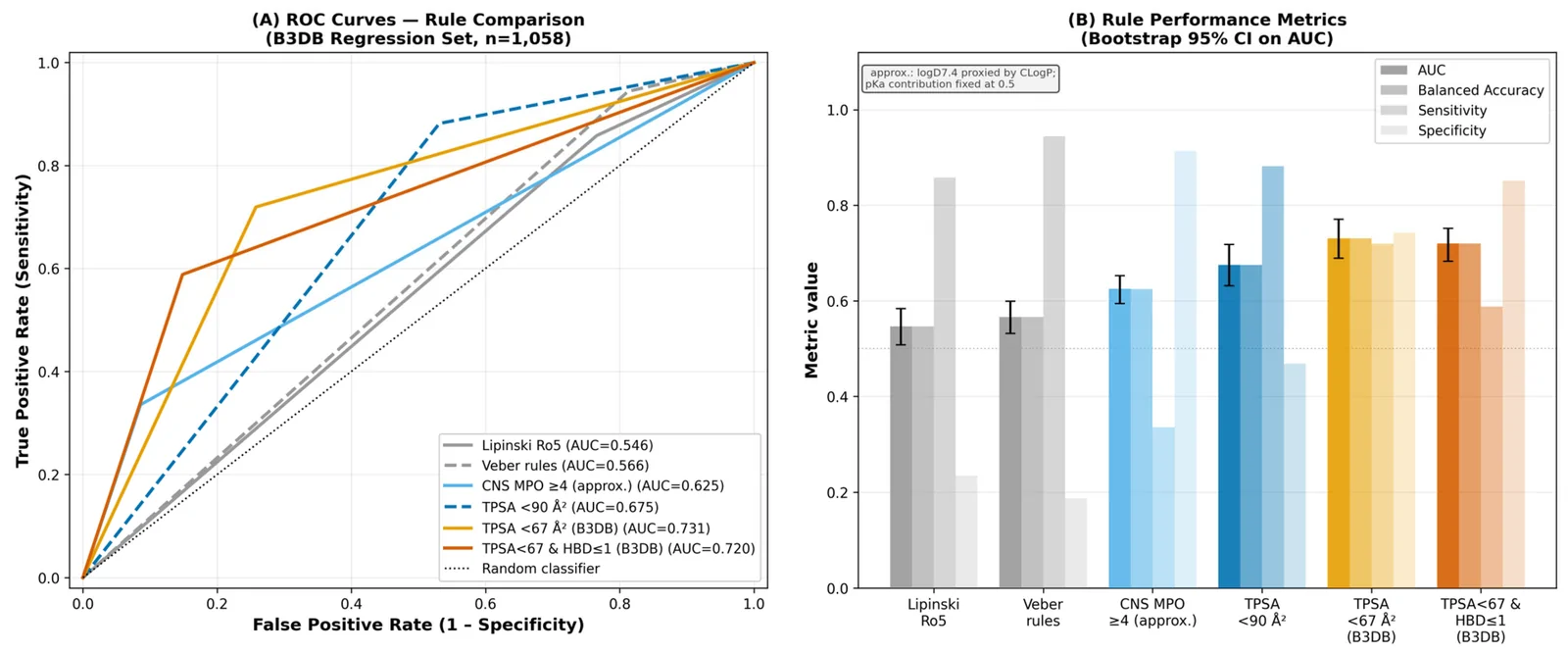
Rule performance comparison on the B3DB regression set (*n* = 1058). (**A**) ROC curves for each rule. The B3DB-derived TPSA < 67 rule (orange) achieved the highest AUC (0.746). (**B**) Bar chart comparing the AUC (with bootstrap 95% CI error bars), balanced accuracy, sensitivity, and specificity across all the rules.

Figure 6 and Table 3 summarize the head-to-head comparison of all rules on the full B3DB dataset (*n* = 1058; 930 BBB+ and 128 BBB−). The B3DB-derived TPSA < 67 rule (AUC = 0.731, balanced accuracy = 0.731) achieved higher discrimination than the approximated CNS MPO ≥ 4 rule (AUC = 0.625; Supplementary Note S1, sensitivity analysis), Lipinski’s Ro5 (AUC = 0.546), and Veber rules (AUC = 0.566). Pairwise bootstrap testing (2000 paired stratified resamples) confirmed that the AUC improvement of TPSA < 67 over all comparators was significant (vs. Lipinski Ro5, *p* < 0.001; vs. Veber rules, *p* < 0.001; vs. CNS MPO (approx.), *p* < 0.001; vs. TPSA < 90, *p* < 0.001; Table 3 footnote). The comparison with CNS MPO should be interpreted considering that this analysis used an approximate implementation based on CLogP and a fixed pKa contribution; the performance using experimentally measured logD and pKa values may differ. The two-feature rule TPSA < 67 AND HBD ≤ 1 achieved the highest precision (96.6% BBB+ when positive), with specificity = 0.852, while maintaining a coverage of 53.5% of compounds (566/1058). In terms of compound coverage, the Veber rules classified 92.8% of compounds (982/1058) as BBB+, representing the least selective approach, whereas CNS MPO (approx.) (52.7% coverage) and TPSA < 67 and HBD ≤ 1 (53.5% coverage) provided a more selective identification of BBB+ compounds.

Notably, the Veber rules achieved high sensitivity (0.944) but very low specificity (0.188), correctly identifying 94% of the BBB-permeable compounds but misclassifying the majority of the BBB-impermeable compounds. Lipinski Ro5 exhibits a similar pattern. In contrast, the B3DB TPSA < 67 AND HBD ≤ 1 rule improves specificity to 0.852 while retaining 96.6% precision among predicted BBB+ compounds, which is more suitable for compound prioritization tasks, where false positives are costly. Additional rule performance metrics are provided in Supplementary Table S2.

#### Use-Case–Specific Rule Selection

The optimal rule depends on the intended applications. For CNS penetration prioritization, where reliable identification of BBB-permeable candidates is desired, the TPSA < 67 AND HBD ≤ 1 rule achieved the highest precision (96.6%, MCC = 0.288, lift = 1.10 relative to the 87.9% class prevalence). For peripheral target design, where reliable identification of BBB-impermeable compounds is required, no single- or two-feature rule evaluated in this study achieved a high negative predictive value (maximum NPV = 0.221 for TPSA < 67 and HBD ≤ 1), reflecting the limitation imposed by the high BBB+ prevalence in the dataset. For CNS exclusion applications, more stringent multiparameter filters or dedicated CNS liability prediction models are recommended; the rules presented here should not be used as the sole basis for CNS exclusion decisions.

### 3.6. Exploratory Scaffold Analysis

**Figure 7:**
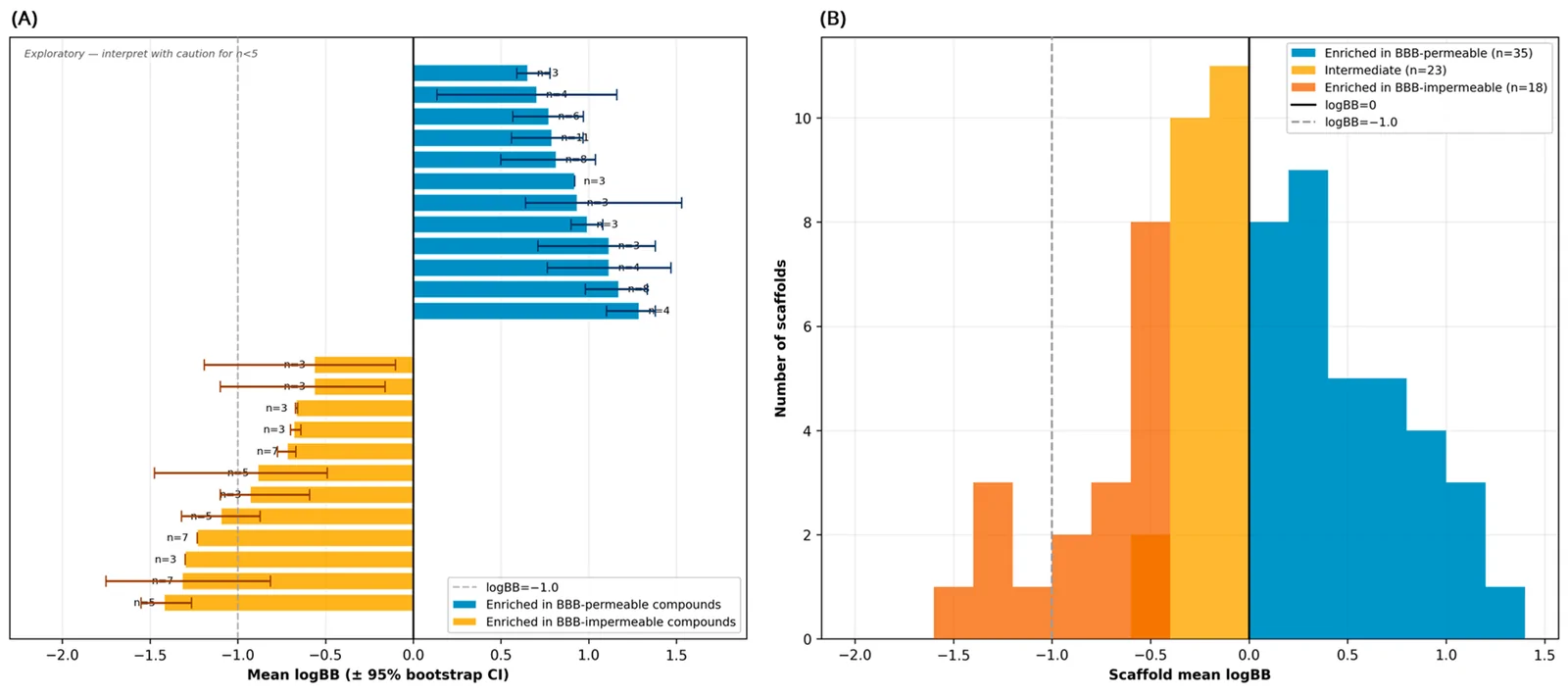
Exploratory scaffold analysis of B3DB compounds. (**A**) Mean logBB with bootstrap 95% confidence intervals (2000 resamples) for the 12 scaffolds most enriched in BBB-permeable compounds (blue) and the 12 scaffolds most enriched in BBB-impermeable compounds (orange) among Murcko scaffolds with *n* ≥ 3. (**B**) Distribution of the mean logBB across 77 qualifying scaffolds. This exploratory analysis describes the scaffold-level logBB patterns; small groups (*n* = 3–5) should be interpreted cautiously. Scaffold SMILES are provided in Supplementary Table S5.

Scaffold analysis is presented as an exploratory characterization of the mean logBB distributions across common structural frameworks. The results should be interpreted descriptively rather than as validated scaffold–activity relationships, particularly for scaffolds with small groups (*n* = 3–5). Murcko scaffold analysis identified 516 unique frameworks among the 1058 B3DB compounds, of which 77 scaffolds had ≥3 representative compounds. Of these, 36 were BBB-permeable (mean logBB ≥ 0), 23 were intermediate (−0.5 ≤ mean logBB < 0), and 18 were BBB-impermeable (mean logBB < −0.5). Figure 7 and Table 4 display the most BBB-permeable and scaffold-enriched BBB-impermeable compounds, respectively.

The most enriched scaffold in BBB-permeable compounds was the piperazinyl-phenyl core (4-phenylpiperazine; mean logBB = +1.29 ± 0.19, *n* = 4), followed by phenothiazine-type systems (mean logBB = +1.17 ± 0.28, *n* = 8) and phenothiazine-piperazine hybrids (+1.12 ± 0.41, *n* = 4). These scaffolds are present in antipsychotics and antihistamines, which are drug classes known for their high CNS penetration. Cyclohexane-based scaffolds also show a high mean logBB (+0.82 ± 0.44, *n* = 8), reflecting the lipophilic and low TPSA characteristics of this core. The most enriched scaffolds in BBB-impermeable compounds included furyl-thiazole systems (mean logBB = −1.42 ± 0.19, *n* = 5), triazolo-nucleoside-type scaffolds (−1.30 ± 0.00, *n* = 3), and oxazolidine-fused tricyclics (−1.32 ± 0.72, *n* = 7). These cores are characterized by a high TPSA, multiple HBA/HBD, and/or charged or zwitterionic character, all of which are in agreement with the threshold analysis above. The furanyl ring, which contributes approximately 13 Å^2^ to the TPSA, appears repeatedly in scaffolds enriched in BBB-impermeable compounds [14].

### 3.7. logBB by Compound Class

**Figure 8:**
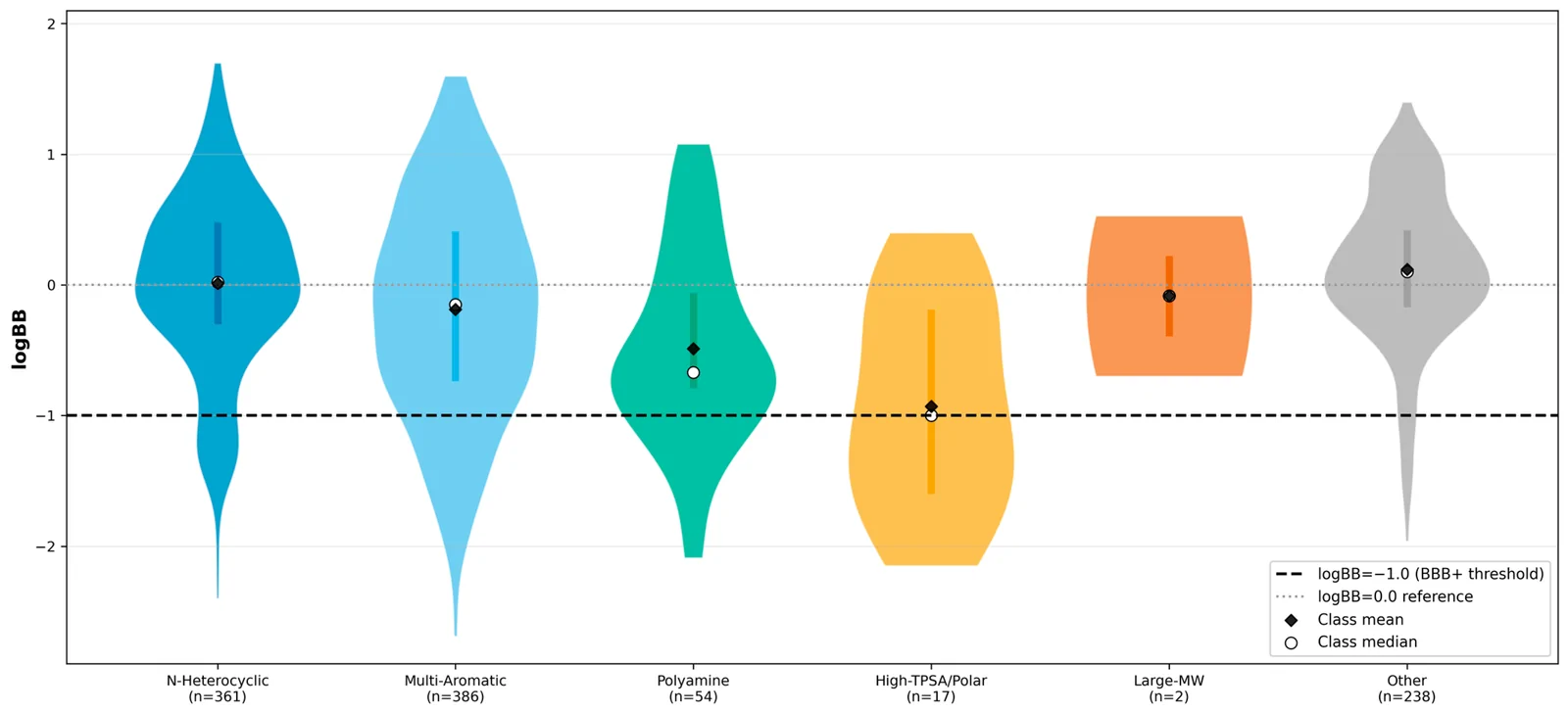
Violin plots of logBB distributions by structural compound class in B3DB (*n* = 1058; Gaussian KDE, Scott bandwidth). Compound classes were assigned hierarchically (non-overlapping by design): N-heterocyclic (*n* = 361), multi-aromatic (*n* = 386), polyamine (*n* = 54), high-TPSA/polar (*n* = 17), large-MW (*n* = 2), and other (*n* = 238). The class definitions are provided in Section 3.7. White circles indicate the class means, and thick horizontal lines indicate the medians. The logBB = −1.0 classification threshold (dashed line) and logBB = 0 reference (dotted line) are shown in the figure.

Violin plots of logBB by compound class (Figure 8) revealed systematic variations consistent with the scaffold results. N-heterocyclic and multi-aromatic compounds showed the widest logBB distributions, reflecting the diverse roles of these scaffolds in CNS and non-CNS drugs. The High-TPSA/Polar class (*n* = 17; TPSA > 100 Å^2^ with HBD ≥ 2) and large-MW compounds (MW > 500 Da; *n* = 2) clusters were substantially below the logBB = −1.0 threshold. Compound classes were defined hierarchically as follows: N-heterocyclic (≥1 nitrogen-containing ring, MW ≤ 350 Da, TPSA ≤ 100 Å^2^, *n* = 361); multi-aromatic (≥2 aromatic rings, *n* = 386); polyamine (≥3 amine nitrogens, *n* = 54); high-TPSA/polar (TPSA > 100 Å^2^ AND HBD ≥ 2, *n* = 17); large-MW (MW > 500 Da, *n* = 2); other (remaining compounds not classified above, *n* = 238). The assignment was hierarchical in the order listed, and the classes were non-overlapping by design.

### 3.8. External Categorical Evaluation of Transportability

**Figure 9:**
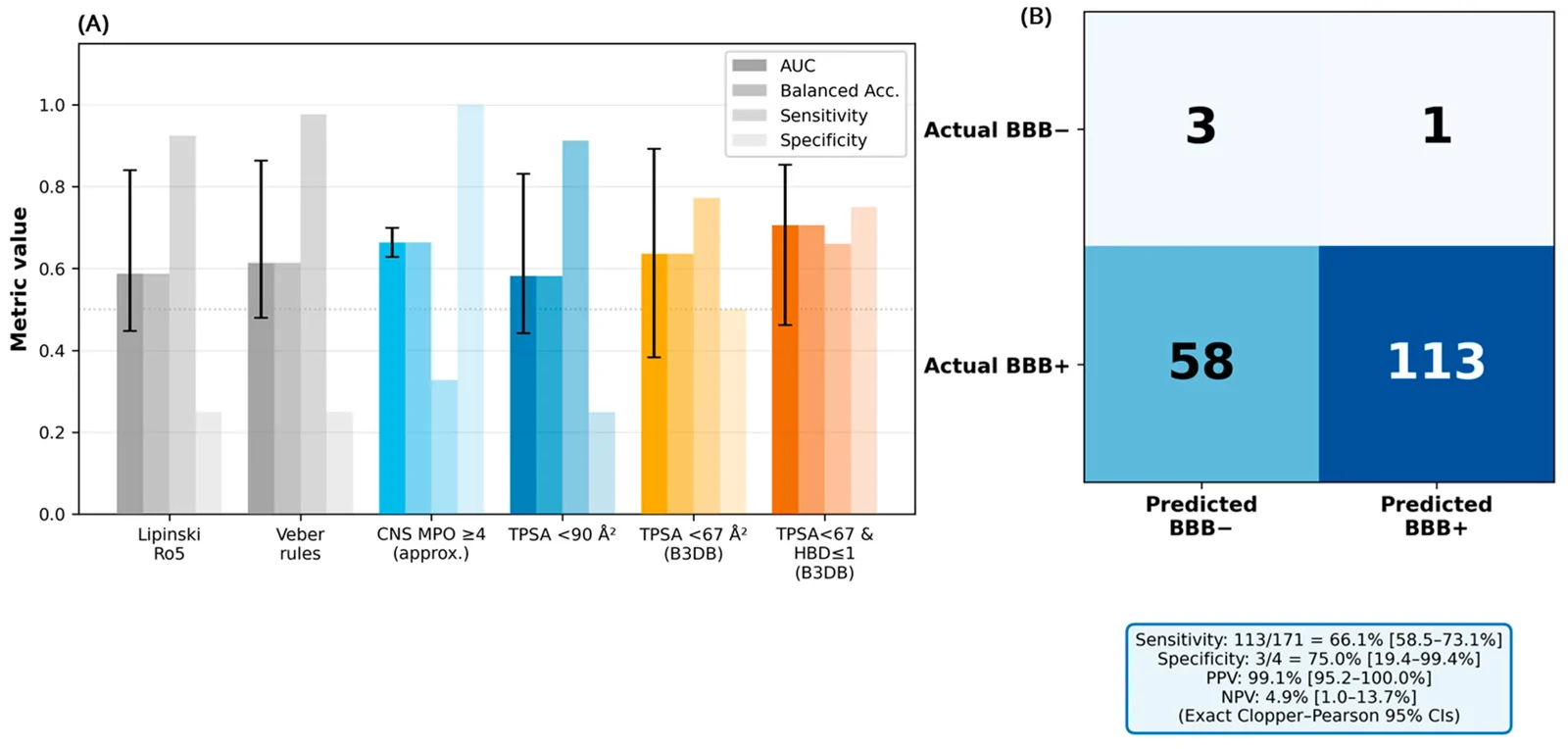
External categorical evaluation of transportability (B3DB external set, *n* = 175; 171 BBB+, 4 BBB−). (**A**) Bar chart showing AUC, balanced accuracy, sensitivity, and specificity for each rule, with bootstrap 95% CI on AUC using stratified resampling while maintaining the 171:4 BBB+− ratio. Specificity estimates should be interpreted cautiously because only four BBB− compounds were available for testing. (**B**) Confusion matrix for the best rule (TPSA < 67 Å^2^ AND HBD ≤ 1): TN = 3, FP = 1, FN = 58, TP = 113. Exact Clopper–Pearson 95% CIs: sensitivity = 66.1% [58.5–73.2%], specificity = 75.0% [19.4–99.4%], PPV = 99.1% [95.2–100%], and NPV = 4.9% [1.0–13.7%]. The limited BBB− representation prevents a reliable assessment of specificity or CNS exclusion utility.

External categorical evaluation used the B3DB external set (*n* = 175; 171 BBB+, 4 BBB−) for the model. The external set was strongly enriched with BBB-permeable compounds (97.7%), making sensitivity the primary metric. The specificity estimates were unreliable owing to the near-complete absence of BBB-impermeable compounds (Figure 9). The full confusion matrix for the TPSA < 67 AND HBD ≤ 1 rule was TP = 113, TN = 3, FP = 1, and FN = 58. Exact Clopper–Pearson 95% confidence intervals were calculated for the sensitivity, specificity, PPV, and NPV. The revised discussion emphasizes that no reliable conclusions regarding specificity, NPV, or CNS exclusion utility can be drawn from this external dataset. External results are now interpreted as an exploratory assessment of transportability to external categorical classifications rather than a quantitative validation of the 66.8 Å^2^ threshold. A balanced external dataset with adequate BBB− representation is required for a definitive assessment of specificity and CNS exclusion utility. The external validation compound data and detailed validation metrics are presented in Supplementary Tables S3 and S4.

## 4. Discussion

### 4.1. TPSA as the Dominant BBB Predictor

Our analysis of 1058 experimental logBB values confirmed that topological polar surface area is the most informative physicochemical descriptor for BBB permeability prediction (AUC = 0.731 [0.689–0.771]), consistent with the SHAP analysis of our companion study [13], in which TopoPSA had the highest mean |SHAP| value (0.318). The data-driven optimal threshold of TPSA = 66.8 Å^2^ is substantially lower than the commonly cited TPSA < 90 Å^2^ used in central nervous system (CNS) drug design guidelines [8]. This tightening has important practical implications: the TPSA < 90 threshold flags 83.9% of compounds as BBB+ (sensitivity = 0.882) but accepts many borderline BBB-impermeable compounds (specificity = 0.469). Restricting to TPSA < 67 improved specificity from 0.469 to 0.742, while maintaining 95.3% precision among predicted positives. This result is consistent with fundamental physicochemical principles; TPSA reflects the desolvation penalty for moving a molecule from aqueous blood into the lipophilic BBB microenvironment [15]. For passive transcellular diffusion, the dominant mechanism for small CNS drugs, this penalty is directly proportional to the polar surface area of the drug. The experimentally optimal threshold (66.8 Å^2^) is well below 90 Å^2^, suggesting that many compounds classified as CNS-eligible by the 90 Å^2^ rule are borderline or BBB-impermeable under passive-diffusion conditions.

### 4.2. Comparison with CNS MPO

The CNS MPO score (Wager et al., 2010; 2016) [8,10] is widely used in industrial CNS drug discovery and integrates six desirability functions into a composite score. The present data show that CNS MPO ≥ 4 (approximately) achieves AUC = 0.625 on B3DB logBB data, which is lower than TPSA < 67 (AUC = 0.731). It should be noted that this comparison involves an approximated CNS MPO score in which logD7.4 was proxied by CLogP, and the pKa desirability contribution was fixed at 0.5 (Supplementary Note S1). The true CNS MPO performance for ionizable compounds computed using the measured logD and pKa values may differ from the values reported here. The CNS MPO score also had a lower sensitivity (0.336) than the simple TPSA rules, indicating that it misclassified the true BBB+ compounds. Several factors may explain these findings. First, CNS MPO was developed using a proprietary dataset of CNS drug candidates, which may not be generalizable to the chemically diverse B3DB datasets. Second, two of the six CNS MPO desirability functions (logD and pKa) could not be directly computed without ionization prediction software, requiring approximation (pKa contribution fixed at 0.5; logD proxied by CLogP), an acknowledged limitation that may underestimate the true CNS MPO performance of ionizable compounds (Supplementary Note S1). Third, the composite nature of CNS MPO, while capturing multidimensional drug-likeness, may smooth the dominant single-feature signal of TPSA. This does not diminish the value of CNS MPO as a holistic tool for CNS-drug design. The score incorporates hERG safety (via CLogP limits), metabolic stability proxies, and CNS drug-likeness criteria, beyond simple BBB crossing. The present data indicate that for the specific task of predicting experimental logBB permeability, simpler TPSA-based rules are more accurate, whereas CNS MPO serves a broader multi-objective optimization role [16].

### 4.3. Two-Feature Rules and Design Implications

The combined rule TPSA < 67 AND HBD ≤ 1 achieved 96.6% BBB+ precision with AUC = 0.720, at the cost of lower sensitivity (0.588) and narrower coverage (53.5% of compounds). In practical terms, this rule advises the bioisosteric replacement of NH/OH groups with N-methylated, fluorinated, or ring-incorporated analogs (to reduce HBD) combined with the optimization of polar functional groups to remain below TPSA = 67 Å^2^. These strategies have been established in CNS drug design but are now quantitatively grounded in 1058 experimental logBB values. Heatmap analysis (Figure 5) provides additional detail. The strongest logBB gradient was along the TPSA axis: crossing from the TPSA 0–40 bin to the 60–80 bin was associated with a reduction of approximately −0.72 logBB units at HBD = 0 (+0.43 vs. −0.29), while each additional HBD at constant TPSA was associated with a smaller reduction of −0.01 to −0.38 logBB units depending on the TPSA range. These quantitative associations are additive rather than synergistic (logistic regression with TPSA × HBD interaction term showed no AUC improvement over the additive model: 0.802 vs. 0.803) and can directly inform lead optimization decisions.

### 4.4. Scaffold Insights

Scaffold analysis identified phenothiazine, piperazinyl-phenyl, and pyrazole-diphenyl cores as highly BBB-permeable (mean logBB +1.1–+1.3) compounds. These scaffolds are characteristic of first- and second-generation antipsychotics, antihistamines, and anxiolytics. Their BBB permeability is in line with the threshold analysis: phenothiazine TPSA is approximately 12.5 Å^2^ and the 4-phenylpiperazine scaffold (one tertiary N-aryl + one secondary N-H) has a TPSA of approximately 15.3 Å^2^ (verified by RDKit)—both scaffold classes are well below the 67 Å^2^ threshold—with a moderate CLogP (2–4). Note that unsubstituted piperazine has TPSA = 24.1 Å^2^ (two secondary amine nitrogens: 2 × 12.03 Å^2^); N-aryl substitution reduces one nitrogen to a tertiary state with a substantially lower PSA contribution (~3.2 Å^2^), yielding the observed scaffold TPSA of ~15.3 Å^2^. Scaffolds enriched in BBB-impermeable compounds are dominated by furan-containing systems, nucleoside-like frameworks, and oxazolidine fusions, which are commonly found in antiviral, antimicrobial, and anticancer agents. The furanyl ring contributes approximately 13 Å^2^ to the TPSA, and combined with amide or carbamate groups in these scaffolds, frequently pushes the total TPSA above 90 Å^2^. Thus, threshold analysis provides a structural rationale for the historically poor CNS penetration of nucleoside analogs: their high intrinsic TPSA from sugar hydroxyl groups and amide functions places them firmly in the BBB-impermeable zone.

### 4.5. Relationship with CNS Medicinal Chemistry Guidelines

Although a TPSA below 90 Å^2^ is frequently cited as favorable for CNS penetration, as seen in the Wager CNS MPO desirability function [8,10] and numerous medicinal chemistry guidelines, several approved CNS drugs have TPSA values between 67 and 90 Å^2^ and achieve adequate brain exposure. Lamotrigine, an antiepileptic present in the B3DB dataset (TPSA = 78 Å^2^, logBB = +0.48), is a well-documented example of a compound above the B3DB-derived threshold yet with high BBB permeability, likely reflecting favorable pKa (pKa ≈ 5.7; predominantly neutral at physiological pH) and low P-glycoprotein efflux liability. Similarly, nitrazepam (TPSA = 84.6 Å^2^, logBB = +0.32) and (R)-pentobarbital (TPSA = 75.3 Å^2^, logBB = +0.12) in B3DB both exceeded the 67 Å^2^ threshold and were classified as BBB+, consistent with their known CNS activity and favorable ionization states. The present analysis is based on experimentally measured logBB values that integrate all transport mechanisms, both passive and active, into a single brain-to-blood ratio. Within this experimental dataset, a threshold of 66.8 Å^2^ offers improved statistical discrimination compared with 90 Å^2^. However, this should be interpreted as a design guideline derived from the observed data distribution, not as a physicochemical boundary that supersedes mechanistic understanding. For compounds where active uptake transporters (e.g., OATPs, LAT1) are anticipated, or where pH-dependent ionization substantially alters the neutral fraction at pH 7.4, passive diffusion assumptions underlying TPSA rules may not be applicable. Practically, the tightened threshold most benefits early-stage CNS lead design, where passive permeability is the dominant hypothesis and transporter involvement remains unclear. The proposed rule TPSA < 67 Å^2^ should be viewed as a first-pass filter: compounds below this threshold have a high prior probability of BBB penetration under passive diffusion, while compounds between 67 and 90 Å^2^ warrant a case-by-case assessment of the ionization state, transporter substrate potential, and measured permeability data. This nuanced interpretation aligns with the broader trend in CNS drug discovery toward mechanistic rather than purely heuristic BBB assessments [1,2].

### 4.6. Limitations and Future Perspectives

The findings of this study should be interpreted within the scope of the available experimental dataset and computational framework employed. The B3DB logBB dataset (*n* = 1058) was compiled from heterogeneous literature sources with varying experimental protocols, species (predominantly rats), and measurement conditions. Such variability may contribute to uncertainties in threshold estimation and descriptor–permeability relationships. Second, although the conventional logBB ≥ −1.0 classification boundary was used, alternative thresholds (e.g., −0.3 or 0.0) may have influenced the optimal decision boundary. However, sensitivity analyses confirmed that TPSA remained the most informative descriptor across the tested cutoffs. Third, descriptor-based rules cannot capture biological factors beyond molecular structure, including active transport mechanisms such as P-glycoprotein efflux, BCRP transport, and other transporter-mediated effects that may influence CNS exposure of the drug. Fourth, the external evaluation dataset (*n* = 175) was strongly enriched in BBB-permeable compounds, limiting the reliability of specificity estimates and preventing definitive assessment of BBB exclusion performance.

Future studies should further evaluate the generalizability of these rules using larger and chemically diverse external datasets with balanced BBB-permeable and BBB-impermeable representation. Additional validation using scaffold-based splitting, experimentally measured logD and pKa values, and unbound brain partition coefficients (Kp, uu, brain) would provide a more mechanistically detailed assessment of CNS penetration. Furthermore, because B3DB integrates brain-to-blood and brain-to-plasma concentration ratios from different studies, harmonized datasets with standardized experimental definitions may further improve the predictive modeling of BBB exposure.

### 4.7. Comparison with the ML Study and Broader Context

This study was a retrospective, secondary computational investigation focused on the derivation and evaluation of interpretable QSPR-based BBB classification rules using an existing experimental logBB database. The analyzed logBB values represent empirical brain-to-blood or brain-to-plasma concentration ratios reported in the source studies and should be interpreted as measures of overall brain exposure rather than as direct permeability coefficients (Papp) or unbound brain exposure parameters (Kp, uu, brain). A recent study [13] demonstrated that gradient boosting on 40 Mordred descriptors achieves AUC = 0.9476 for BBB classification—considerably higher than the AUC = 0.746 of the best single-feature rule reported here. The AUC difference reflects the well-known trade-off between interpretability and predictive power. The decision tree rules of the present work sacrifice approximately 0.20 AUC units in exchange for complete transparency and zero computational overhead: a medicinal chemist can apply TPSA < 67 AND HBD ≤ 1 by inspection of any structure drawing without the use of computational software. These two complementary approaches serve different purposes. The gradient boosting model of the reported work [13] is optimal for virtual screening, computational filtering of large compound libraries, and quantitative logBB predictions. The rules of the present study are optimal for medicinal chemistry decision-making during analog design, SAR rationalization, and the teaching/communication of BBB design principles. Both approaches were validated using the same B3DB experimental data to ensure internal consistency of the results.

## 5. Conclusions

Using 1058 experimental logBB measurements from the B3DB database, we derived and internally evaluated data-driven structural rules for predicting BBB permeability, with a preliminary external categorical assessment of transportability. The key findings are:

1. The topological polar surface area emerged as the most predictive physicochemical property for BBB permeability (AUC = 0.731 [0.689–0.771]), with an optimal B3DB-derived threshold of 66.8 Å^2^, which is substantially lower than the commonly applied 90 Å^2^ limit for BBB permeability.
2. A simple two-feature rule (TPSA < 67 Å^2^ AND HBD ≤ 1) achieved 96.6% BBB+ precision with AUC = 0.720 and specificity = 0.852, outperforming the approximated CNS MPO ≥ 4 (AUC = 0.625), Lipinski Ro5 (AUC = 0.546), and Veber rules (AUC = 0.566) on the experimental data.
3. Decision trees (depth = 4) explained 36.0% of the logBB variance (R^2^ = 0.360, RMSE = 0.603; 5-fold CV AUC = 0.780 ± 0.044) and produced interpretable IF-THEN rules with TPSA, SLogP, and autocorrelation descriptors as primary decision variables.
4. The scaffold enriched in BBB-permeable compounds included phenothiazine (mean logBB = +1.17 ± 0.28), piperazinyl-phenyl (+1.29 ± 0.19), and pyrazole-diphenyl (+1.12 ± 0.36) cores, while the scaffold enriched in BBB-impermeable compounds included furyl-thiazole (−1.42 ± 0.19), oxazolidine-tricyclic (−1.32 ± 0.72), and triazolo-nucleoside (−1.30 ± 0.00) frameworks.
5. The B3DB-derived TPSA < 67 threshold should be applied as a design guideline for passive-diffusion–dominated CNS penetration. Compounds with TPSA values between 67 and 90 Å^2^ require a case-by-case mechanistic assessment, particularly regarding their ionization state and active transport potential.

These data-driven rules offer complementary approaches spanning interpretable heuristics to computational tools and provide experimentally grounded design guidance for early-stage CNS drug discovery.

## Figures and Tables

**Table 1:** IF–THEN rules extracted from the regression decision tree (depth = 4), ordered by mean predicted logBB. TPSA and TopoPSA are numerically identical in this dataset (Pearson r = 1.000); their co-occurrence reflects the implementation differences between RDKit and Mordred rather than independent information. GATS2c = Geary autocorrelation lag-2 charge-weighted; MATS1v = Moran autocorrelation lag-1 vdW-volume weighted; MATS2v = Moran autocorrelation lag-2 vdW-volume weighted; MATS1c = Moran autocorrelation lag-1 charge-weighted; AXp-0d = atomic composition index degree 0; fMF = molecular framework fraction. LabuteASA = Labute approximate surface area (Å^2^). The descriptor definitions are provided in the Supplementary Material (Tables S1–S11).

Rule (IF → THEN)	*n* (Train)	Mean logBB	Category
TPSA > 61.7 AND TopoPSA > 84.3 AND MATS2v ≤ 0.08 AND nBase > 1.50	17	−1.374	Strongly BBB−
TPSA > 61.7 AND TopoPSA > 84.3 AND MATS2v > 0.08 AND fMF > 0.41	17	−1.085	Strongly BBB−
TPSA > 61.7 AND TopoPSA > 84.3 AND MATS2v ≤ 0.08 AND nBase ≤ 1.50	88	−0.852	BBB−
TPSA ≤ 61.7 AND TPSA > 36.3 AND LabuteASA ≤ 90.1 AND AXp-0d ≤ 0.74	15	−0.745	BBB−
TPSA > 61.7 AND TopoPSA ≤ 84.3 AND GATS1c ≤ 1.77 AND MATS1c ≤ −0.43	65	−0.628	BBB−
TPSA > 61.7 AND TopoPSA > 84.3 AND MATS2v > 0.08 AND fMF ≤ 0.41	46	−0.414	Borderline
TPSA > 61.7 AND TopoPSA ≤ 84.3 AND GATS1c ≤ 1.77 AND MATS1c > −0.43	42	−0.166	Borderline
TPSA ≤ 61.7 AND TPSA > 36.3 AND LabuteASA ≤ 90.1 AND AXp-0d > 0.74	15	−0.099	Borderline
TPSA ≤ 61.7 AND TPSA ≤ 36.3 AND SLogP ≤ 2.10 AND MATS1v ≤ −0.03	43	−0.008	Borderline
TPSA > 61.7 AND TopoPSA ≤ 84.3 AND GATS1c > 1.77	20	+0.040	BBB+
TPSA ≤ 61.7 AND TPSA > 36.3 AND LabuteASA > 90.1 AND nBondsD > 0.50	144	+0.042	BBB+
TPSA ≤ 61.7 AND TPSA ≤ 36.3 AND SLogP ≤ 2.10 AND MATS1v > −0.03	20	+0.354	BBB+
TPSA ≤ 61.7 AND TPSA ≤ 36.3 AND SLogP > 2.10 AND GATS2c ≤ 1.06	31	+0.365	BBB+
TPSA ≤ 61.7 AND TPSA > 36.3 AND LabuteASA > 90.1 AND nBondsD ≤ 0.50	60	+0.393	BBB+
TPSA ≤ 61.7 AND TPSA ≤ 36.3 AND SLogP > 2.10 AND GATS2c > 1.06	117	+0.772	Strongly BBB+

**Table 2:** Physicochemical property statistics for BBB+ (*n* = 930) and BBB− (*n* = 128) compound classes (B3DB *n* = 1058). The B3DB-derived optimal thresholds and single-feature AUC values are shown. All BBB+ vs. BBB− differences were significant (*p* < 0.001, Mann–Whitney U test, two-sided).

Property	BBB+ Mean ± SD	BBB− Mean ± SD	BBB+ Median [IQR]	BBB− Median [IQR]	Optimal Threshold	AUC [95% CI]	Cliff’s δ [95% CI]	*p* (Holm)
TPSA (Å^2^)	53.9 ± 41.4	100.9 ± 62.7	48.2 [28.1–70.9]	87.3 [66.8–117.5]	≤66.8	0.746 [0.708–0.784]	−0.595 [−0.671, −0.516]	<0.001
MW (Da)	311.7 ± 152.5	384.6 ± 199.2	304.6 [224.4–385.5]	341.8 [252.3–456.2]	≤442	0.583 [0.544–0.622]	−0.226 [−0.331, −0.123]	<0.001
CLogP	2.82 ± 1.72	1.89 ± 2.62	2.9 [1.7–4.0]	1.9 [1.0–3.3]	>2.3	0.617 [0.564–0.670]	+0.246 [+0.133, +0.354]	<0.001
HBD	1.18 ± 1.22	2.48 ± 2.23	1.0 [0.0–2.0]	2.0 [1.0–3.0]	≤1	0.699 [0.651–0.746]	−0.488 [−0.571, −0.409]	<0.001
HBA	3.52 ± 2.36	5.85 ± 3.75	3.0 [2.0–5.0]	5.0 [4.0–7.0]	≤4	0.673 [0.619–0.726]	−0.458 [−0.552, −0.363]	<0.001
nRotB	3.96 ± 3.10	5.54 ± 3.77	4.0 [1.0–6.0]	5.0 [3.0–8.0]	≤7	0.596 [0.542–0.650]	−0.254 [−0.364, −0.152]	<0.001

Mann–Whitney U tests were performed between BBB+ (*n* = 930) and BBB− (*n* = 128) groups. Effect sizes were calculated using Cliff’s delta, with 95% bootstrap confidence intervals (2000 resamples). Negative delta values indicated higher distributions in the BBB− group. *p*-values were adjusted using Holm’s correction. The exact U statistics are provided in Supplementary Table S2.

**Table 3:** Head-to-head comparison of BBB permeability rules on the B3DB regression set (*n* = 1058; 930 BBB+ and 128 BBB−). Bootstrap 95% CI on AUC (2000 resamples, seed = 42). BalAcc = balanced accuracy = (Sensitivity + Specificity)/2. MCC = Matthews correlation coefficient. AUPRC = area under the precision-recall curve. Lift = Precision/class prevalence (87.9%). All values were verified using the B3DB dataset.

Rule	AUC [95% CI]	BalAcc	F1	Sens	Spec	Precision	NPV	MCC	AUPRC	Macro-F1	Lift	Coverage
Prevalence classifier (always BBB+)	0.500 [—]	0.500	0.935	1.000	0.000	0.879	0.000	0.000	0.879	0.500	1.000	1.000
LR baseline (TPSA + CLogP) [test set only, *n* = 212]	0.684 [0.569–0.793] test set only	—	—	—	—	—	—	—	—	—	1.001	0.972
Lipinski Ro5	0.546 [0.508–0.584]	0.546	0.874	0.858	0.234	0.891	0.185	0.084	0.889	0.541	1.013	0.847
Veber rules	0.566 [0.532–0.599]	0.566	0.918	0.944	0.188	0.894	0.316	0.166	0.893	0.577	1.017	0.928
CNS MPO ≥ 4 (approx. *)	0.625 [0.594–0.652]	0.625	0.498	0.336	0.914	0.966	0.159	0.177	0.908	0.385	1.099	0.305
TPSA < 90 Å^2^	0.675 [0.631–0.718]	0.675	0.902	0.882	0.469	0.923	0.353	0.311	0.918	0.652	1.050	0.839
TPSA < 67 Å^2^ (B3DB)	0.731 [0.689–0.771]	0.731	0.820	0.719	0.742	0.953	0.267	0.319	0.932	0.606	1.084	0.664
TPSA < 67 and HBD ≤ 1 (B3DB)	0.720 [0.683–0.752]	0.720	0.731	0.588	0.852	0.966	0.222	0.288	0.930	0.541	1.099	0.535

* CNS MPO (approx.): logD7.4 proxied by CLogP; pKa desirability contribution fixed at 0.5. The pKa assumption does not change the binary AUC of the approximated implementation (see Supplementary Note S1). Pairwise bootstrap comparison (2000 resamples, stratified) vs. TPSA < 67 Å^2^ (B3DB): vs. Lipinski Ro5 *p* < 0.001; vs. Veber rules *p* < 0.001; vs. CNS MPO (approx.) *p* < 0.001; vs. TPSA < 90 Å^2^ *p* < 0.001; vs. TPSA < 67 and HBD ≤ 1 *p* = 0.055 (not significant). LR baseline evaluated on held-out test set only (*n* = 212); all other rules on full dataset (*n* = 1058). The prevalence classifier (always predicting BBB+) provided a lower bound for the AUC.

**Table 4:** Exploratory Murcko scaffold analysis of mean logBB distributions in B3DB. Scaffolds were categorized as enriched in BBB-permeable or BBB-impermeable compounds based on the observed mean logBB distributions. Bootstrap 95% confidence intervals (2000 resamples) were provided for the mean logBB estimates.

Category	Core/Scaffold Type	*n*	Mean logBB	SD
Enriched in BBB-permeable compounds	4-Phenylpiperazine	4	+1.29	0.19
Enriched in BBB-permeable compounds	Phenothiazine	8	+1.17	0.28
Enriched in BBB-permeable compounds	Phenothiazine-piperazine	4	+1.12	0.41
Enriched in BBB-permeable compounds	Pyrazole-diphenyl	3	+1.12	0.36
Enriched in BBB-permeable compounds	Phenylpiperazine-cyclohexyl	3	+0.99	0.09
Enriched in BBB-permeable compounds	Benzylpyridine	3	+0.94	0.51
Enriched in BBB-permeable compounds	Cyclohexane	8	+0.82	0.44
Enriched in BBB-permeable compounds	Indolo-hexahydro	6	+0.77	0.27
Enriched in BBB-impermeable compounds	Furyl-thiazole	5	−1.42	0.19
Enriched in BBB-impermeable compounds	Triazolo-oxazolidine	3	−1.30	0.00
Enriched in BBB-impermeable compounds	Oxazolidine-tricyclic	7	−1.32	0.72
Enriched in BBB-impermeable compounds	Thiazolyl-nucleoside	5	−1.30	0.00
Enriched in BBB-impermeable compounds	Oxetane-triazole	5	−1.10	0.31
Enriched in BBB-impermeable compounds	Cyclohexene	5	−0.89	0.64
Enriched in BBB-impermeable compounds	Furanyl-pyrimidine	5	−0.72	0.05
Enriched in BBB-impermeable compounds	Tetrahydrofuryl-pyrimidine	7	−0.72	0.07

This exploratory analysis describes scaffold-level logBB distributions rather than validated scaffold–activity relationships. Scaffolds with small group sizes (*n* < 5) should be interpreted with caution. SD = 0.00 for triazolo-oxazolidine (*n* = 3) and thiazolyl-nucleoside (*n* = 5) reflect convergence of logBB values after rounding to two decimal places; exact unrounded values are provided in Supplementary Table S5.

## Data Availability

The original contributions presented in this study are included in the article/supplementary material. Further inquiries can be directed to the corresponding author.

## Supplementary Materials

The following supporting information can be downloaded at: https://www.mdpi.com/article/10.3390/pharmaceutics18080967/s1, Table S1. B3DB Regression Dataset—Complete Compound Data (n = 1058). Table S2. Rule Metrics. Table S3. External Validation—B3DB External Classification Set (n = 175). Table S4. B3DB External Validation Set—All 175 Compounds. Table S5. Scaffold Analysis. Table S6. Mean logBB by TPSA × H-Bond Donor Bins (Figure 6 Source Data). Table S7. Decision Tree IF-THEN Rules—All Leaf Nodes (depth = 4). Table S8. Mordred/RDKit Descriptor Definitions. Table S9. Cutoff_Sensitivity. Table S10. Tanimoto_Similarity. Table S11. Scaffolds_n5plus. Note S1. CNS MPO Score Approximation and Sensitivity Analysis.
